# Supplementary figures and images for: DNABERT-based explainable lncRNA identification in plant genome assemblies
Source: Comput Struct Biotechnol J. 2023 Nov 17;21:5676–85. doi: 10.1016/j.csbj.2023.11.025 (PMC10696397; doi:10.1016/j.csbj.2023.11.025)

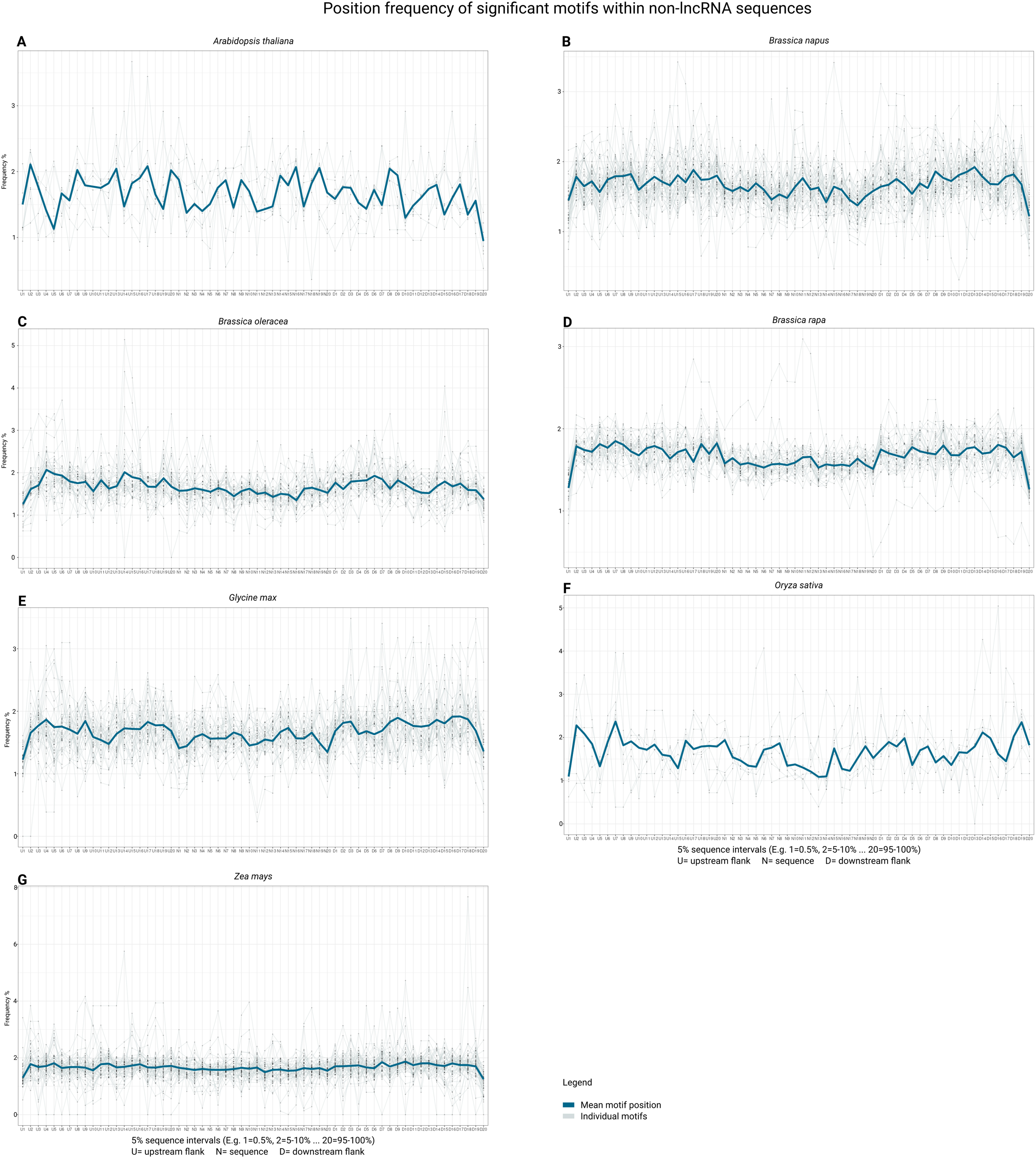

Supplement: Supplementary file 6 — Supplementary materialSupplementary Figure 1: Position frequency of significant motifs within the non-lncRNA sequences. [file mmc6.jpg]
